# Contemporary strategies for repeat ablation of atrial fibrillation: a European Heart Rhythm Association survey

**DOI:** 10.1093/europace/euaf231

**Published:** 2025-09-22

**Authors:** Sergio Conti, Ante Anic, Giulio Conte, Christian-H Heeger, Jarkko Karvonen, Andreas Metzner, Mark T Mills, Martina Nesti, Diego Penela, Rui Providencia, Laurent Roten, Martin H Ruwald, Kostantinos Vlachos, Maura M Zylla, Kyoung-Ryul Julian Chun

**Affiliations:** Department of Internal Medicine, Division of Cardiology, Section of Clinical Cardiac Electrophysiology, University of Iowa Health Care, The Carver College of Medicine, University of Iowa, 200 Hawkins Dr, Iowa City, IA 52242, USA; Department for Cardiovascular Diseases, University Hospital Center Split, Split, Croatia; Division of Cardiology, Cardiocentro Ticino Institute, Ente Ospedaliero Cantonale, Lugano, Switzerland; Faculty of Biomedical Sciences, USI, Lugano, Switzerland; German Center for Cardiovascular Research (DZHK), Partner Site Hamburg/Kiel/Lübeck, Lübeck, Germany; Department of Rhythmology, University Heart Center Lübeck, University Hospital Schleswig-Holstein, Lübeck, Germany; Heart and Lung Center, Helsinki University Hospital and University of Helsinki, Helsinki, Finland; University Heart & Vascular Center, University Medical Center Hamburg-Eppendorf, Hamburg, Germany; Department of Cardiology, Liverpool Heart & Chest Hospital, Thomas Drive, Liverpool, UK; Department of Cardiology, Fondazione Toscana Gabriele Monasterio, Pisa, Italy; Arrhythmology Department, Humanitas Research Hospital IRCCS, Rozzano, Milan, Italy; Institute of Health Informatics Research, University College London, London, UK; Barts Heart Centre, St. Bartholomew’s Hospital, Barts Health NHS Trust, London, UK; Department of Cardiology, Inselspital, Bern University Hospital, University of Bern, Bern, Switzerland; Division of Electrophysiology, Department of Cardiology, Herlev-Gentofte Hospital, Denmark; IHU LIRYC ANR-10-IAHU-04, Centre Hospitalier Universitaire Bordeaux, Bordeaux, France; Department of Cardiology, Heidelberg Center of Heart Rhythm Disorders, Medical University Hospital, Heidelberg, Germany; Cardioangiologisches Centrum Bethanien, Agaplesion Markus Krankenhaus, Frankfurt am Main, Germany

**Keywords:** Atrial fibrillation, Repeat procedures, Arrhythmia recurrence, Ablation strategy, Ablation technologies

## Abstract

**Aims:**

Pulmonary vein isolation (PVI) is the cornerstone of atrial fibrillation (AF) ablation. However, the optimal strategy during repeat ablation is not clear. This European Heart Rhythm Association (EHRA) survey aims to assess real-world ablation strategies in patients undergoing repeat AF ablation.

**Methods and results:**

A 25-item questionnaire was distributed among healthcare professionals via EHRA between 22 May and 21 June 2024. Of the 211 respondents from 43 countries, 58.1% of respondents planned a redo after multiple symptomatic recurrences of atrial arrhythmias. Most repeat procedures (68.0%) are performed within 3 months after the decision for re-intervention. 3D mapping and radiofrequency (RF) catheters with contact force (CF) sensing are the most common modality used for repeat ablation. In patients with more than one pulmonary vein (PV) reconnection, most commonly reisolation of the PVs plus individualized substrate-based ablation is performed (62.2%). When empirical ablation is performed, the most common targets include cavotricuspid isthmus (22.5%), posterior wall isolation (20.7%), left atrial roofline (16.1%), anterior line (12.9%), superior vena cava (8.6%), and vein of Marshall (8.6%). In patients without PV reconnection at repeat procedure, substrate mapping/individualized ablation is the preferred strategy (77.9%). No additional right atrial ablation beyond the CTI was reported. The majority of respondents (60.7%) consider rate control after ≥3 failed ablations.

**Conclusion:**

Real-world strategies for repeat AF ablation show significant variability. 3D mapping and CF-guided RF ablation are commonly utilized. Re-PVI and substrate-based ablation are the predominant approaches. However, the optimal strategy beyond durable PVI remains to be further evaluated.

## Graphical Abstract

**Graphical Abstract:**
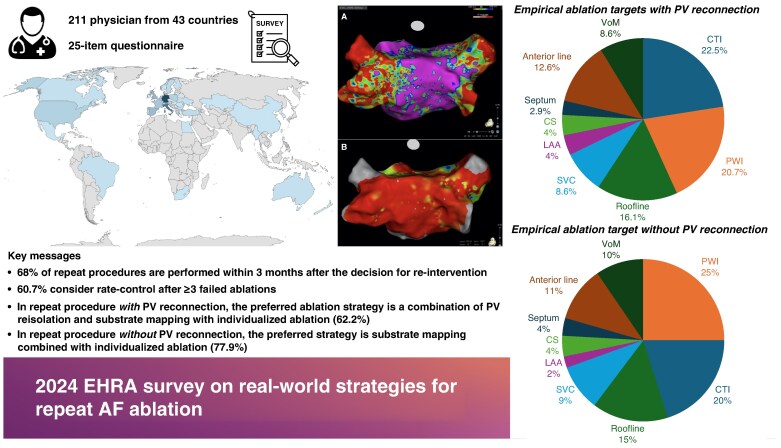

What's new?

- The most common strategy employed for the index procedure was pulmonary vein isolation (PVI) only in 81.2% of cases. Despite recent technological advancements, radiofrequency ablation (RFA), particularly with contact force (CF) sensing catheters, was the predominant technology used for PVI. Cryoballoon ablation was used by 31% of respondents and multispline/multielectrode pulsed field ablation by 9%.
- The majority of respondents considered RFA with CF to be the technology associated with the best PVI durability.
- Patterns of atrial arrhythmia recurrence—atrial fibrillation (AF) or atrial tachycardia/atrial flutter (AT/AFLs)—did not significantly influence the decision to proceed with a repeat ablation procedure. When AF was the predominant recurrence pattern, 79.3% of physicians opted for repeat ablation, and similarly, 84.1% made the same choice when AT/AFL was the main recurrence pattern.
- Pulmonary vein reconnection (89.3%), the presence of significant comorbidities (75.6%), and the delivery of wide antral PVI during the index procedure (42.6%) were considered to favour AF recurrence. Additional linear ablations and complex fractionated atrial electrogram ablation performed during the index procedure were considered as predisposition for AFL/AT recurrence (84.4% and 61.1%, respectively).
- The most common mapping strategies adopted during redo AF ablation included substrate mapping in sinus rhythm to delineate low-voltage areas in the left atrium (88.5%) and PV mapping (83.6%). In 73.7% of cases, the mapping strategy combined both approaches. Less frequently, AF electrogram-based mapping (22.9%) and substrate mapping in sinus rhythm for low-voltage areas in the right atrium (12.3%) were utilized.

## Introduction

Pulmonary vein isolation (PVI) is the cornerstone of any atrial fibrillation (AF) ablation procedure.^1,2^ The pivotal challenge of AF ablation is achieving durable isolation of the pulmonary veins (PVs),^3^ which is often hindered by the resumption of electrical conduction in previously isolated PVs.^4,5^ The non-negligible rate of PV reconnection is primarily due to non-transmural and/or non-contiguous ablation lesions, and this is a major factor contributing to the recurrence of arrhythmia after AF ablation. This observation has driven the assessment of novel ablation strategies and modalities that enhance lesion transmurality and PVI durability.^6^ Point-by-point radiofrequency (RF) ablation has been the mainstay of AF ablation for decades and is still the most adopted technology. Several technologies and catheter designs have been developed as alternative approaches to facilitate PVI, such as the cryoballoon, RF balloon, LASER balloon, and pulsed field ablation (PFA).^3,7–13^ These devices allow fast, simple, and more reproducible procedures making them appealing options for first-time AF ablation attempts. However, despite technological advancements, PV reconnection remains a common finding in patients experiencing AF recurrence. Furthermore, there is a lack of clear strategies for repeat AF ablation beyond PVI.

## Aim

The European Heart Rhythm Association Scientific Initiatives Committee (EHRA SIC) conducted a survey to investigate the current management approaches for patients with recurrent AF and which repeat ablation strategies are adopted among European and non-European electrophysiologists.

## Methods

### Online questionnaire

The questionnaire was distributed using SurveyMonkey with the support of the EHRA SIC. The online-based questionnaire consisted of single- and multiple-choice questions aimed at assessing the technologies and strategies for index PVI ablation, their impact on repeat AF ablation strategies, factors influencing physicians’ decisions to perform repeat procedures, specific strategies for repeat ablations with and without PV reconnection, and strategies adopted when rhythm control has failed. The full questionnaire, provided in the Supplementary material online, *Appendix S1*, was reviewed and the final version approved by all investigators. Participation in the survey was voluntary, anonymous, and compliant with GDPR regulations. The electronic link to the survey was sent to EHRA members via the EHRA newsletter, made accessible for 4 weeks from 22 May and 21 June 2024, and also promoted through social media.

### Statistical analysis

Continuous variables are presented as mean ± standard deviation or as median and interquartile range. Categorical variables are expressed as numbers and percentages. Test for normality of the distribution was assessed visually. Questionnaires with relevant missing data were excluded on a case-by-case basis. All statistical analyses were performed with SPSS v25 (IBM Corp.), and figures were created using Excel (Microsoft).

## Results

### Section 1: demographics of respondents

**Figure 1:**
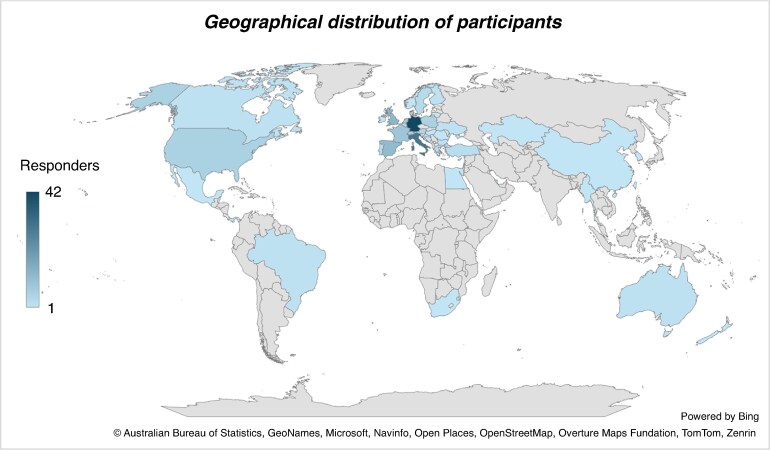
Geographical representation of the distribution of the survey’s respondents.

**Figure 2:**
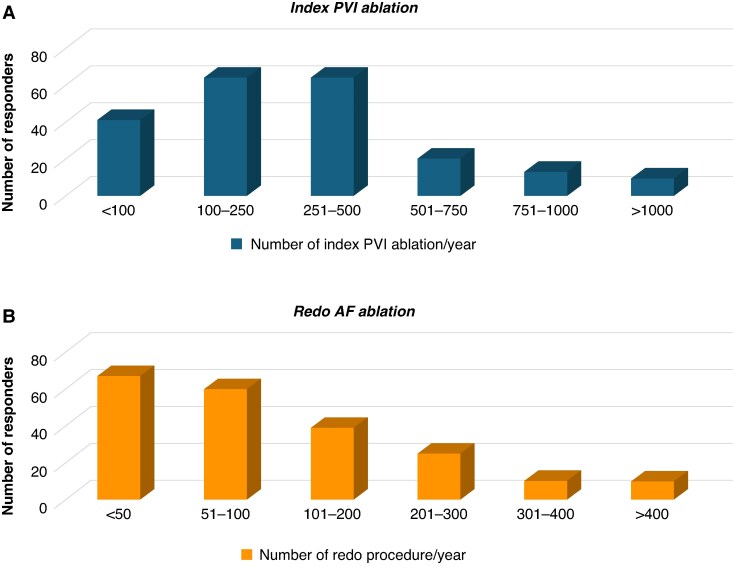
(*A*) Number of index PVI procedure/year. (*B*) Number of redo procedure/year.

A total of 211 participants completed the survey. The majority (59.6%) worked at university hospitals, followed by public hospitals (29.4%), private hospitals (8.6%), and office or research facilities (2.4%). Respondents came from 43 different countries, with a predominant practice in Europe (60.5%), followed by the Asia-Pacific region (18.6%), the Americas (11.6%), and Africa and the Middle East (9.3%) (*Figure 1*). Most respondents (60.7%) worked in medium-high volume electrophysiology (EP) centres that perform between 100 and 500 index AF ablation procedures annually, 19.4% worked in lower volume centres performing <100 index PVI procedures per year, and 19.9% in high or very-high volume centres that perform >500 ablations (*Figure 2A*). Regarding redo AF procedures, the majority of respondents states to perform <100 redo AF procedures per year (60.2%), while 18.5% performed between 101 and 200 per year, and 21.3% performed >200 procedures annually (*Figure 2B*). Most of respondents (57.8%) worked in a facility with on-site cardiothoracic surgical services.

### Section 2: strategies and technologies for index atrial fibrillation ablation procedure

**Figure 3:**
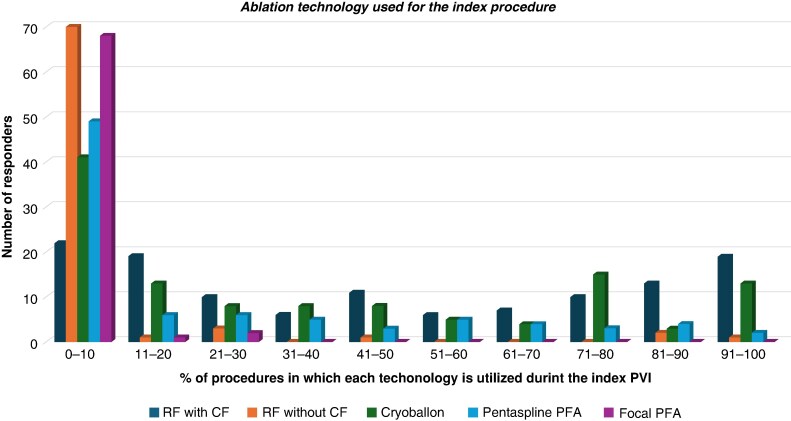
Ablation technologies used for the index procedure. Each bar represents the percentage of procedures in which each of the technology evaluated in this survey is used during the index PVI. RF, radiofrequency; CF, contact force; PFA, pulsed field ablation.

The most commonly employed strategy for the index procedure was PVI only, utilized by 81.2% of physicians. In contrast, 16.6% of physicians performed additional individualized substrate-based ablation, and 2.2% included additional right atrial ablation. Radiofrequency ablation (RFA), particularly with contact force (CF) sensing catheters, was the predominant technology used for achieving PVI. Specifically, 42% of respondents indicated that they used RFA with CF in most cases for the index procedure, while cryoballoon ablation (CBA) was used by 31% and multispline multielectrode PFA catheters by 9%. The full distribution of answers, including other technologies used, is depicted in *Figure 3*.

**Figure 4:**
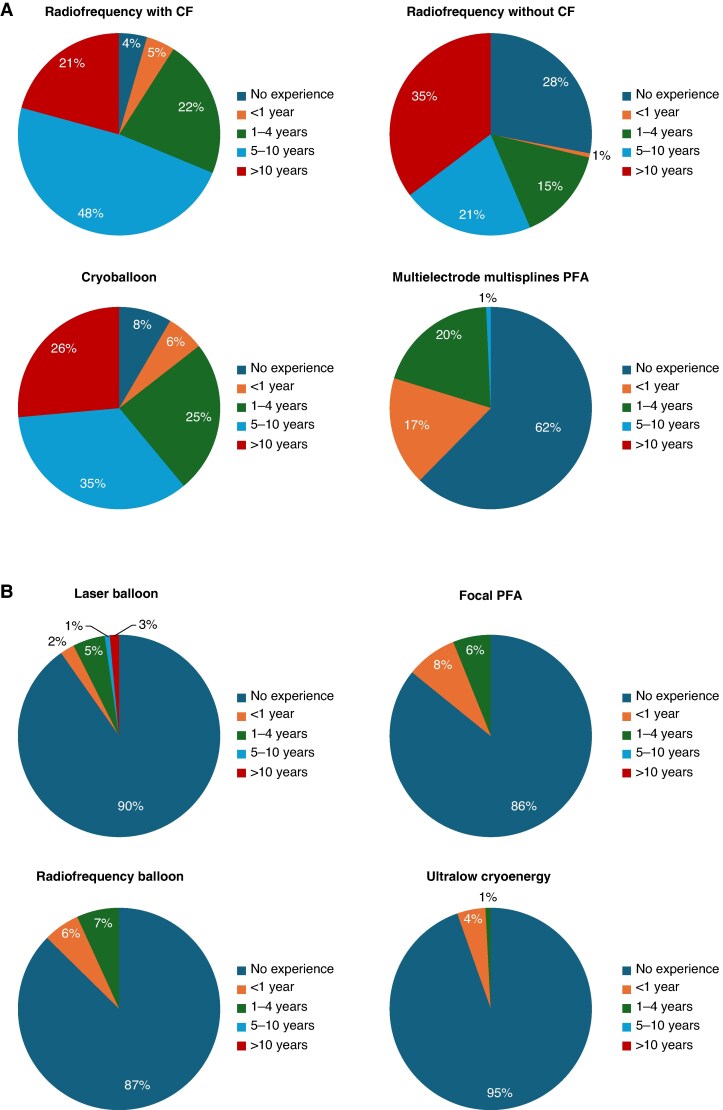
(*A* and *B*) Operator experience with each technology.

Most physicians reported having long-term experience (>5 years) with RFA with CF sensing catheters, RFA without CF, and CBA. Although 62% of physicians did not have any experience with PFA at the time of the survey, 17.1% reported short-term experience of less than 1 year, and 18.6% had experience ranging from 1 to 4 years. More than 85% of respondents lacked experience with other ablation technologies, including laser balloon ablation, RF balloon ablation, focal PFA catheter, and ultralow cryoablation (*Figure* *4A* and *B*).

**Figure 5:**
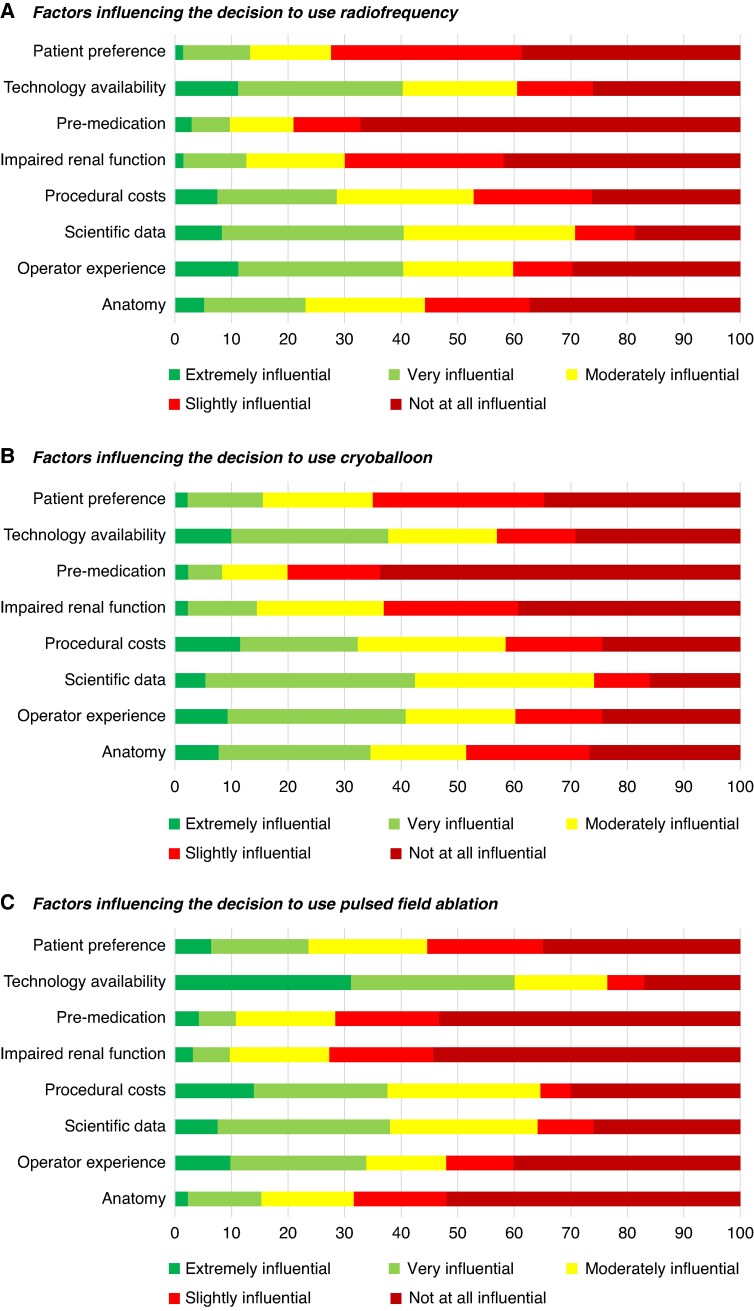
(*A*) Factors influencing the decision to use RF. (*B*) Factors influencing the decision to use cryoballoon. (*C*) Factors influencing the decision to use PFA (*Table 1*).

To assess the factors influencing the choice of the three primary ablation modalities (RFA, CBA, PFA) for index PVI, respondents rated various factors on a 5-point Likert scale, from ‘Extremely influential’ to ‘Not at all influential’. The factors included (i) left atrial anatomy, (ii) operator experience, (iii) scientific data, (iv) procedural costs, (v) renal function, (vi) pre-existing medications, (vii) technology availability, and (viii) patient preference. The three most influential factors for selecting RF were scientific data, operator experience, and technology availability. For CBA, the key factors were scientific data, operator experience, and procedural costs. For PFA, the most influential factors were technology availability, scientific data, and procedural costs (*Figure 5A–C*).

In terms of perceived effectiveness, RFA with CF was considered the technology associated with the best PVI durability, with 46.6% of preferences, followed by CBA at 21.1%, and multispline/multielectrode PFA at 18%. The second choice of technology preference was CBA, which garnered 35.7% of preferences, followed by RFA with CF at 25.4%, and multispline/multielectrode PFA at 15.9%. Finally, RFA without CF received 33.3% of preferences, followed by CBA with 35.7%, which consistently gained 24.7% of preferences.

### Section 3: strategies and technologies for repeat atrial fibrillation ablation

The impact of the following factors on the decision to perform a repeat AF ablation was assessed on a 5-point Likert scale, ranging from ‘Redo justified’ to ‘Redo not justified’. The factors considered included (i) AF as the main arrhythmia recurrence pattern, (ii) atrial tachycardia (AT) or atrial flutter (AFL) as the main arrhythmia recurrence pattern, (iii) presence of untreated comorbidities, (iv) specific reasons encountered during the index procedure, (v) recurrence that occurred only during the blanking period as defined by the latest guidelines^1^, and (vi) low burden of any atrial arrhythmia (AF/AT/AFL) recurrence. Patterns of atrial arrhythmia recurrence—whether AF or AT/FLs—did not significantly influence the decision to proceed with a redo AF ablation. In fact, when AF was the predominant recurrence pattern, 79.3% of physicians opted for repeat ablation. Similarly, 84.1% made the same choice when AT/AFL was the main recurrence pattern. Only 6.3% of physicians felt that a repeat AF ablation was justified in the case of untreated comorbidities, while 30.1% deemed it was unjustified, and 54.8% of respondents were neutral. Specific reasons faced during the index procedure, such as complex anatomy or difficult transseptal access, did not appear to affect the decision for a repeat procedure. Here, only 6.3% of respondents decided against a second procedure, and 25.4% considered a redo, while 59.5% were neutral. When arrhythmia recurrence occurred solely during the blanking period, 76.2% of physicians chose not to perform repeat AF ablation. Finally, in cases with a low arrhythmia burden, only 9.6% of physicians would consider carrying out a repeat AF ablation.

Regarding the timing of repeat AF ablation for recurrences beyond the blanking period, more than half of the respondents (58.1%) indicated they would plan a repeat procedure after multiple symptomatic AF/AT recurrences. Additionally, 21.6% would do so after asymptomatic recurrence that required cardioversion, 14.4% after the first symptomatic AF/AT recurrence, and 5.9% after any AF/AT recurrence, including asymptomatic episodes. The most common waiting period from the decision to perform repeat ablation until the execution of the procedure is 3 months (45.9% of responses). Shorter waiting periods (6 weeks or less) are unusual, as are longer waiting periods (6 months or more), which accounted for 22.1% and 27.8% of the responses, respectively.

When performing a redo AF ablation, the most commonly used technology is RFA with CF. Approximately 56% of physicians employ it in every case, while 73.2% use it in more than 90% of their cases. A high-density multielectrode mapping catheter is the preferred technology for checking entrance and exit block (53.6%), followed by standard spiral/circular mapping (28.7%) and ablation catheter (17.7%).

Respondents were also asked which factors they believe are more likely to drive AF recurrence versus AFL/AT recurrence. They felt that PV reconnection is more likely to favour AF recurrence (89.3%), as well as the presence of significant comorbidities (75.6%). Additional linear ablations and complex fractionated atrial electrogram (CFAE) ablation performed during the index procedure were considered as predisposition for AFL/AT recurrence (84.4% and 61.1%, respectively). Furthermore, the delivery of wide antral PVI during the index procedure was considered to favour AFL/AT recurrence by 42.6% of respondents. The most common mapping strategies adopted during repeat AF ablation include substrate mapping in sinus rhythm to delineate low-voltage areas in the left atrium (88.5%) and PV mapping (83.6%). In 73.7% of cases, mapping strategy combined both approaches. Less frequently, AF electrogram-based mapping (22.9%) and substrate mapping in sinus rhythm for low-voltage areas in the right atrium were utilized (12.3%).

### Section 4: ablation strategies in redo procedures with pulmonary vein reconnection

**Figure 6:**
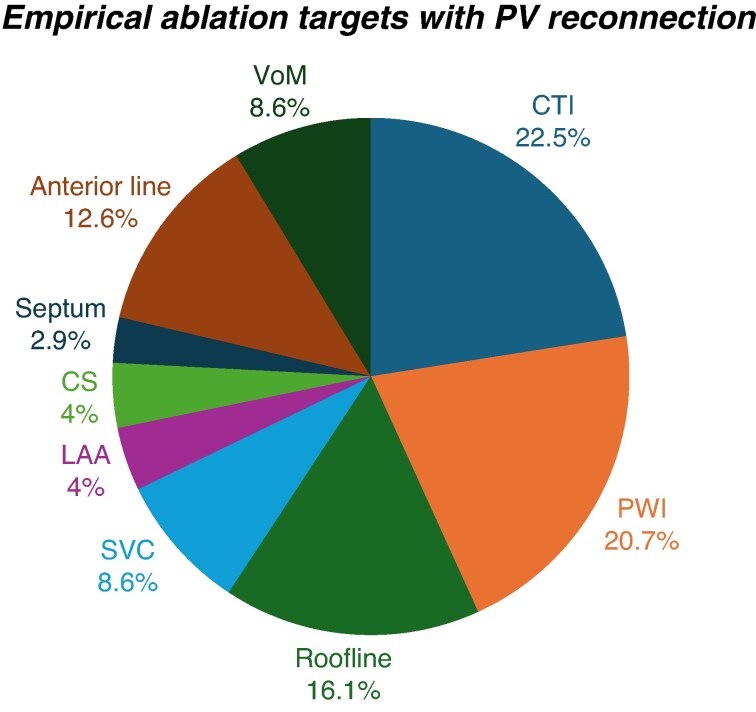
Graphical representation of empirical ablation targets in case there is evidence of PV reconnection. CTI, cavotricuspid isthmus; PWI, posterior wall isolation; SVC, superior vena cava; LAA, left atrial appendage; VoM, vein of Marshall; CS, coronary sinus.

In patients with more than one PV reconnection at repeat ablation, the preferred ablation strategy was a combination of PV reisolation and substrate mapping with individualized ablation (62.2%). This was followed by PV reisolation only (45.4%), PV reisolation plus ablation of non-PV triggers (22.7%), PV reisolation plus more antral ablation (21.8%), and finally PV reisolation with empirical target ablation without any documentation of substrate or firing (15.1%). If empirical ablation beyond PVI was performed, the most common targets included the cavotricuspid isthmus (CTI), followed by posterior wall isolation (PWI), and left atrial roofline ablation (*Figure 6*). The majority of respondents (70.3%) indicated that they would not change their strategy if only one PV was reconnected. Among those that would modify their approach, most (80%) opted for PV reisolation only. If all PVs were reconnected, 60.7% of physicians would maintain their original strategy. Among those who would change strategy, 86.7% choose PV reisolation only.

### Section 5: ablation strategies in redo procedures without pulmonary vein reconnection

**Figure 7:**
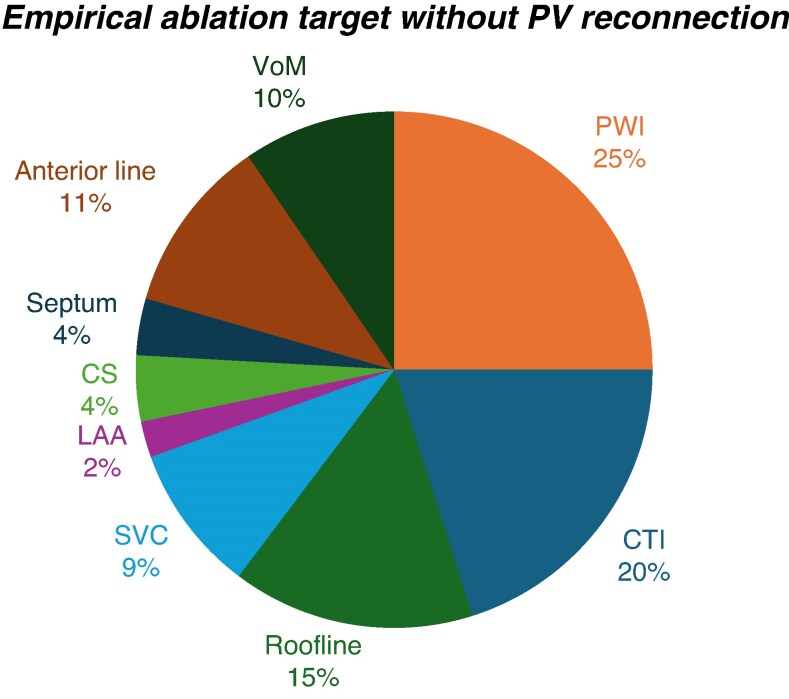
Graphical representation of empirical ablation targets in case there is no evidence of PV reconnection. CTI, cavotricuspid isthmus; PWI, posterior wall isolation; SVC, superior vena cava; LAA, left atrial appendage; VoM, vein of Marshall; CS, coronary sinus.

In patients with no PV reconnection during repeat ablation, the preferred strategy was substrate mapping combined with individualized ablation (77.9%). This was followed by non-PV trigger ablation (34.7%) and then more antral ablation or empirical target ablation (15.2%). If empirical ablation was performed, the most common strategy was PWI, followed by CTI and roofline ablation (*Figure 7*). One in 10 (10.2%) physicians would conclude the procedure without performing further ablation, and 8.5% would re-establish antiarrhythmic drugs (AADs).

### Section 6: failed rhythm control

If rhythm control was not achieved despite catheter ablation and/or AADs, the most common strategy employed was pharmacological rate control, utilized by 46.1% of respondents. This was followed by pacemaker implantation and atrioventricular node ablation (‘pace and ablate’) at 35.9%. Other options included referral for surgical ablation (8.5%), transfer to a different centre (2.6%), and alternative approaches, which account for 5.2% (including hybrid ablation, 1.7%, and various strategies depending on the age of the patients, 2.6%). The majority of respondents (60.7%) considered rate control after three or more failed ablations to be a suitable option, while a third found it acceptable (33.3%) after two failed ablations.

Interestingly, 6 out of 10 respondents (60.6%) worked in a hospital that performs surgical AF ablation. Despite this, 66.1% of these physicians never referred a patient for surgical AF ablation, with 18.3% doing so only after three or more failed ablations and 13.9% after two failed ablations.

## Discussion

The results of this survey show a heterogeneous management of patients undergoing repeat AF ablation. These findings mainly concern the most controversial issues, including factors influencing the decision to perform a repeat ablation, the timing of repeat ablation, the identification of ablation targets outside the PVs, and the overall ablation strategy. At the time of this survey, most physicians had more than five years of experience with RFA using CF sensing catheters, RFA without CF, and CBA. Notably, 62% of physicians reported having no experience with PFA. It is likely that variations in geographical and economic environments have affected access to the latest technologies.

As previously shown, many AF relapses are due to PV reconnection.^14^ In these cases, repeat procedures focus on identifying and closing PV reconnection gaps.^15^ The rate of durable PVI among patients experiencing AF recurrences varies, ranging from 15 to 79% after an initial CBA and 26 to 62% following RFA.^16^ Among patients enrolled in the CIRCA-DOSE study, 15% underwent a repeat procedure, with PV reconnection identified in 90.4% of patients, with no significant differences between the three groups (RF–CBA 4–CBA 2). Overall, 44.3% of PVs were reconnected.^17^ Data from the repeat ablation guided by the CLOSE–PVI protocol, which used rigorous strategy based on the delivery of contiguous and optimized RF lesions, indicated that all PVs remained isolated in 62% of patients.^18^

Recent studies, including the MANIFEST-REDO trial and the multicentre EU-PORIA registry have provided new insights on the topic of PVI durability. In the MANIFEST-REDO study, only 44% of patients who experienced clinical recurrence after an initial PFA procedure had all PVs durably isolated. Among those with recurrences, 29% had one PV reconnected, 16% had two, 9% had three, and 1% had all four PVs reconnected.^19^ The EU-PORIA registry demonstrated a high single-procedure success rate with an excellent safety profile and short procedure times in a real world.^20^ Data from repeat ablation procedures in patients who experience arrhythmia recurrence after initial PVI performed using PFA showed a durable isolation in 71% of the PVs during the redo procedure, and 38% of all patients had durable isolation of all PVs.^21^ Another study indicated that less than half of the patients undergoing repeat procedures maintained durable PVI, with reconnection gaps preferentially occurring at the anterior aspects of the right-sided PVs.^22^ Although PFA has unquestionably several advantages over previous energy forms, the durability of PVI with currently available PFA modalities in real-world setting is lower than initially anticipated. The overall rate of PV reconnection during repeat procedures is comparable to that observed with other energy sources. Noteworthy, regarding lesion durability using PFA on the posterior wall, encouraging data were reported by Kueffer *et al*. Durable PWA was found in 85% of patients with only minor lesion regression. This finding is remarkable considering that such low reconnection rates were achieved using the first clinically available PFA catheter, which was not designed for PWI and did not offer intrinsic 3D mapping integration.^23^

In this survey, when addressing patients with documented PV reconnection, the preferred ablation strategy primarily focused on PV reisolation, which was the only strategy adopted in 45.4% of the responses. However, the most common approach, utilized in 62.2% of cases, involved substrate mapping combined with individualized ablation on top of PV reisolation. In patients who showed no PV reconnection during repeat ablation, the preferred strategy was substrate mapping combined with individualized ablation in 77.9% of patients. These findings suggest that, despite the advent and availability of new technologies, the strategies adopted for repeat AF ablation remain heterogeneous across centres and countries.

The optimal ablation strategy for patients experiencing clinical recurrences of AF despite having durable PVI remains unknown. In this context, the retrospective, multicentre PARTY-PVI study presented the largest cohort of patients undergoing repeat ablation for AF in which durable PVI was documented.^16^ This study evaluated the clinical outcomes associated with single or combination strategies during repeat AF ablation procedures. The strategies investigated were linear-based, EGM-based, trigger-based, and PV-based ablation. The findings indicated that none of these techniques proved superiority in enhancing arrhythmia-free survival, and outcomes were similar for both paroxysmal and persistent AF. Additionally, left atrial dilatation was identified as a significant predictor of atrial arrhythmia recurrence.

Among extra-PV targets, the role of left atrial appendage (LAA) as AF trigger remains uncertain. Previous studies have shown that LAA isolation can improve clinical outcomes for patients with AF who do not respond to PVI. However, electrical isolation of the LAA, along with a loss of its mechanical function, may lead to an increased incidence of LAA thrombus and thromboembolism, even with appropriate anticoagulation therapy.^24^ Recently, the multicentre prospective randomized ASTRO-AF study compared LAA isolation to substrate modification in patients with drug-refractory persistent AF, despite durable PVI after a first ablation procedure. However, this study found no significant advantage of cryoballoon-guided LAA isolation over ablation of low-voltage areas for patients with AF who had durable PVI.^25^

Another relevant aspect from this survey that is worth mentioning is the timing of repeat AF ablation. Despite the latest recommendations regarding the duration of the blanking period,^1^ the most common waiting time for repeat AF ablation was 3 months. Earlier procedures falling within the blanking period or immediately after were uncommon. However, the scheduling of repeat AF ablation procedures may also be influenced by waiting list dynamics. Noteworthy, with the expected larger adoption of PFA, it is also important to consider the results of the multicentre prospective study published by Mohanty *et al*.^26^ This study reassessed the duration of the blanking period after PFA and documented a high risk of late recurrence for patients who experienced early recurrence during the second and third months of the blanking period. They concluded that the blanking period after PFA could potentially be reduced to just the first month following the procedure.

The surprisingly low percentage of referrals for surgical AF ablation is noteworthy. This finding is particularly difficult to explain, especially since more that more than half of the physicians who responded to the survey work in a facility with on-site cardiothoracic surgical services. A possible explanation may be the absence of a dedicated surgical AF ablation programme or a structured collaboration between surgeons and electrophysiologists.

Finally, recent encouraging results have emerged from the application of artificial intelligence (AI) in this field. Tailored cardiac ablation guided by an AI-based algorithm has been shown to significantly improve long-term outcomes compared to PVI alone in patient with persistent AF, as demonstrated in the randomized TAILORED-AF trial.^27^ The application of an AI-based algorithm in repeat AF ablation has not yet been explored but may offer a novel alternative for treating these patients in the future.

### Limitations

The voluntary nature of this survey introduces selection bias and raises concerns about whether these findings represent a realistic picture of the current practice. Although the survey was distributed using different methods, such as the EHRA members’ newsletter and various social media platforms, this limitation is inherent to the survey’s nature, as it tends to attract respondents with specific interests, strong opinions, or peculiar characteristics that align with the survey topic. This may create a self-selected, skewed sample that does not necessarily reflect the diverse viewpoints of the general EP community. As a result, the survey’s findings may present a narrower picture of the real-world scenario.

## Conclusions

A standardized approach to patients undergoing a repeat AF ablation is lacking. While the primary goal of any AF ablation is to achieve durable PVI, the optimal strategy for managing AF recurrence in patients with documented PVI remains unclear. Further multicentre, randomized trials are necessary to clarify this uncertainty and guide future clinical practice.

**Table 1:** Factors that can potentially influence the decision to use a certain technology to perform the first-time AF ablation

	Extremely influential	Very influential	Moderately influential	Slightly influential	Not at all influential
Radiofrequency					
Anatomy	5.3	17.9	20.9	18.6	**37**.**3**
Operator experience	11.2	29.1	19.4	10.4	**29**.**9**
Scientific data	8.3	**32**.**3**	30.1	10.5	18.8
Procedural costs	7.5	21.1	24.1	21	**26**.**3**
Impaired renal function	1.5	11.2	17.3	27.9	**42**.**1**
Pre-medication	3	6.7	11.2	11.9	**67**.**2**
Technology availability	11.2	**29**.**1**	20.1	13.4	26.2
Patient preference	1.5	11.9	14.2	33.6	**38**.**8**
Cryoballoon					
Anatomy	7.7	**26**.**9**	16.9	21.6	26.9
Operator experience	9.3	**31**.**5**	19.2	15.4	24.6
Scientific data	5.4	**37**	31.5	10	16.1
Procedural costs	11.5	20.8	**26**.**1**	17	24.6
Impaired renal function	2.3	12.3	22.3	23.8	**39**.**3**
Pre-medication	2.3	6.1	11.5	16.2	**63**.**9**
Technology availability	10	27.7	19.2	13.9	**29**.**2**
Patient preference	2.3	13.2	19.4	30.3	**34**.**8**
PFA					
Anatomy	2.3	13	16.3	16.3	**52**.**3**
Operator experience	9.8	23.9	14.1	12	**40**.**2**
Scientific data	7.6	**30**.**4**	26.1	9.8	26.1
Procedural costs	14.1	23.6	26.9	5.3	**30**.**1**
Impaired renal function	3.2	6.5	17.5	18.4	**54**.**4**
Pre-medication	4.3	6.5	12.1	10.8	**66**.**3**
Technology availability	**31**.**2**	29	16.1	6.5	17.2
Patient preference	6.5	17.2	20.8	20.4	**45**.**6**

In bold, the highest percentage for each item.

## Supplementary Material

euaf231_Supplementary_Data

## Data Availability

The data underlying this article will be shared on reasonable request to the corresponding author.
