# Supplementary material for: Contemporary strategies for repeat ablation of atrial fibrillation: a European Heart Rhythm Association survey
Source: Europace. 2025 Sep 22;27(11):euaf231. doi: 10.1093/europace/euaf231 (PMC12602869; doi:10.1093/europace/euaf231)
Supplement: euaf231_Supplementary_Data [file euaf231_supplementary_data.pdf]

Survey on strategies for repeat ablation for Atrial Fibrillation

Introduction

The EHRA Scientific Initiatives Committee is conducting a survey on strategies for repeat ablation for Atrial Fibrillation (AF).

A durable Pulmonary Vein Isolation (PVI) is the cornerstone of AF ablation procedures. Although several technologies and energy sources have been introduced into clinical practice, PV reconnection remains a common finding in patients who experience AF recurrence. Moreover, there are no clear indication for performing repeat ablations in AF.

This EHRA survey seeks to assess the existing approaches to AF redo procedures among electrophysiologists.

The survey consists of 35 questions and will take you less than 10 minutes to complete. Your participation will be considered anonymous. You have the right to end your participation in this survey at any time.

Survey on strategies for repeat ablation for Atrial Fibrillation

GDPR Disclaimer

1. Your participation is anonymous.
We will not disclose your identity to any third party.
You have the right to end this survey at any time.

We comply with the European General Data Protection Regulation (GDPR) 2016/679. Any personal data processed in connection with this survey will be treated confidentially and only used by the ESC for the purposes of market research and not for promotion. Survey results will be kept for a maximum of 48 months for analysis and quality control purposes. We take all reasonable care to prevent any unauthorized access to your personal data. We respect your privacy and your right to access, modify, or remove your personal data. At any time, you can ask to know what personal data is being held. If you have any questions about data protection or require further information, please contact our data protection officer (DPO) at dpo@escardio.org.

You have the right to end your participation in this survey at any time.

Please confirm that you have read the above and agree to participate in this survey.

- ◯ Yes
- ◯ No

SECTION 1: Participant centre characteristics

2. In which country is your centre based?

3. Primary working environment

- Academic hospital
- Non-academic hospital
- Private hospital / clinic
- Office-based practice
- Research institution / Research university
- Other (please specify)

4. How many **first do** AF ablation procedures are performed in your center/year?

- <100
- 100-250
- 251-500
- 501-750
- 751-1000
- >1000

5. How many **redo** AF ablation procedures are performed in your center/year?

- <50
- 51 – 100
- 101 –200
- 201 – 300
- 301 – 400
- > 401

6. Do you have cardiac surgery onsite in your institution?

- Yes
- No

SECTION 2: Strategies/technologies for first AF Ablation

7. What ablation technology do you use in your EP lab for first AF ablation? Please indicate the (%) for each of the ablation technologies (total 100%).

Radiofrequency current (RFC) with Contac Force (CF) ____

RFC without CF ____

Cryoballoon ____

Multielectrode / multisplines Pulsed Field Ablation (PFA) ____

Focal PFA ____

Others ____

8. When performing a **first** PVI for AF, what influences your decision to use RFC in your EP lab ?

	Not at all influential	Slightly influential	Moderately influential	Very influential	Extremely influential
Anatomy of the left atrium	○	○	○	○	○
Operator experience	○	○	○	○	○
Scientific data	○	○	○	○	○
Costs of the procedure	○	○	○	○	○
Impaired renal function	○	○	○	○	○
Pre-medication anti-thrombotic + anti-coagulation	○	○	○	○	○
System/technology availability	○	○	○	○	○
patient preference	○	○	○	○	○

9. When performing a **first** PVI for AF, what influences your decision to use Single shot like Cryoballoon in your EP lab ?

	Not at all influential	Slightly influential	Moderately influential	Very influential	Extremely influential
Anatomy of the left atrium	○	○	○	○	○
Operator experience	○	○	○	○	○
Scientific data	○	○	○	○	○
Costs of the procedure	○	○	○	○	○
Impaired renal function	○	○	○	○	○
Pre-medication anti-thrombotic + anti-coagulation	○	○	○	○	○
System/technology availability	○	○	○	○	○
patient preference	○	○	○	○	○

10. When performing a **first** PVI for AF, what influences your decision to use PFA (multielectrode/multipsline) in your EP lab ?

	Not at all influential	Slightly influential	Moderately influential	Very influential	Extremely influential
Anatomy of the left atrium	○	○	○	○	○
Operator experience	○	○	○	○	○
Scientific data	○	○	○	○	○
Costs of the procedure	○	○	○	○	○
Impaired renal function	○	○	○	○	○
Pre-medication anti-thrombotic + anti-coagulation	○	○	○	○	○
System/technology availability	○	○	○	○	○
patient preference	○	○	○	○	○

11. What is your strategy in a **first** AF ablation procedure ?

- ○ PVI only
- ○ PVI + additional **empirical** ablation
- ○ PVI + additional **individualized** ablation

12. How long is your personal experience with ... ?

	no experience	<1 year	1 – 4 years	5 – 10 years	> 10 years
RF with CF	○	○	○	○	○
RF without CF	○	○	○	○	○
Cryoballoon	○	○	○	○	○
Multielectrode multisplines PFA or single shot multielectrode PFA	○	○	○	○	○
Focal PFA	○	○	○	○	○
LASER balloon	○	○	○	○	○
RF balloon	○	○	○	○	○
Ultralow cryo	○	○	○	○	○
Other - please specify below	○	○	○	○	○

Please specify if you selected the 'other' answer option above

SECTION 3: Strategies/technologies for repeat AF ablation procedure

13. How do you assess the following factors to justify a repeat AF ablation ?

	no redo	neutral	redo
AF as arrhythmia recurrence	○	○	○
AT/AFL as arrhythmia recurrence	○	○	○
Non-treated comorbidities (e.g. Adeps)	○	○	○
Special reasons occurred during index procedure (e.g. difficult transseptal puncture)	○	○	○
Recurrence only in blanking period	○	○	○
Low AF/ATs burden	○	○	○

14. When do you plan to perform a **redo** AF ablation beyond blanking period ?

- [ ] After the first symptomatic AF/AT recurrence
- [ ] After multiple symptomatic AF/AT recurrences
- [ ] After any (incl. asymptomatic) AF/AT recurrence
- [ ] After asymptomatic recurrence but when cardioversion is necessary

15. How long is the wait time in your center for a redo AF/AT ablation procedure

- ○ < 6 weeks
- ○ 6 weeks
- ○ 3 months
- ○ 6 months
- ○ 6 – 12 months
- ○ >12 months

16. When performing a redo AF, what ablation technology do you use in your EP lab? Please give a percentage from 0 to 100% for each of the device/ablation technology listed below.

RF with CF	
RF without CF	
Cryoballoon	
Multielectrode multisplines PFA or single shot multielectrode PFA	
Focal PFA	
Others	

17. Which catheter do you use to check for entrance and for exit block?

- [ ] Spiral/circular mapping
- [ ] Ablation catheter
- [ ] High Density mapping catheter
- [ ] Please indicate specific technology

18. Please select and rank **your** top three ablation technologies associated with durable PVI

	gold medal	silver medal	bronze medal	NA
RF with CF	○	○	○	○
RF without CF	○	○	○	○
Cryoballoon	○	○	○	○
Multielectrode / multisplines PFA	○	○	○	○
Focal PFA	○	○	○	○
LASER balloon	○	○	○	○
RF balloon	○	○	○	○
Ultralow cryo	○	○	○	○

19. What do you think favors AF or AT as mechanism of recurrence?

	Favours AF	Neutral	Favours AT
PV reconnection	○	○	○
Linear ablation during index procedure	○	○	○
Wider antral PV isolation during index procedure	○	○	○
Previous CFAE ablation	○	○	○
Comorbidities (e.g. obesity, OSAS, uncontrolled hypertension)	○	○	○

20. What are the mapping strategies driving your ablation in repeat procedures ?

- ☐ Substrate mapping in SR to delineate low voltage areas in left atrium
- ☐ Substrate mapping in SR to delineate low voltage areas in right atrium
- ☐ Time to LAA measurement
- ☐ PV mapping
- ☐ AF electrogram based mapping
- ☐ MRI analysis to determine fibrotic areas

SECTION 4: Specific ablation strategies in Redo procedures with PV reconnection

21. If PVs are reconnected, what is your strategy? (multiple answers)

- [ ] Re-isolate the PVs, then STOP
- [ ] Re-isolate the PVs plus non-PV trigger and ablation
- [ ] Re-isolate the PVs plus substrate mapping and individualized ablation
- [ ] Re-isolate the PVs plus GP ablation
- [ ] Re-isolate the PVs plus ostial potential ablation
- [ ] Re-isolate the PVs plus a more antral ablation
- [ ] Re-Isolate the PVs and ablate empirical targets (see below)
- [ ] Post ablation Re-establish AAD

22. **Empirical ablation targets beyond PVI**, select all that apply:

- [ ] I do not ablate empirical targets
- [ ] CTI
- [ ] PWI
- [ ] Roofline
- [ ] SVC
- [ ] LAA
- [ ] CS
- [ ] Septum
- [ ] Anterior line
- [ ] Ligament /vein of Marshall

23. If **one single** PV is reconnected, do you change your strategy ?

- ○ Yes
- ○ No

24. If yes, what is your strategy ?

- ○ Re-isolate the PV, **then STOP (no more options allowed)**
- ○ Re-isolate the PV plus non-PV trigger ablation
- ○ Re-isolate the PV plus substrate mapping and individualized ablation
- ○ Re-isolate the PV plus ostial potential ablation
- ○ Re-isolate the PV plus a more antral ablation
- ○ Re-Isolate the PV and ablate empirical targets (see below)
- ○ Post ablation Re-establish AAD

25. Empirical ablation targets beyond PVI, select all that apply:

- [ ] CTI
- [ ] PWI
- [ ] Roofline
- [ ] SVC
- [ ] LAA
- [ ] CS
- [ ] Septum
- [ ] Ligament / vein of Marshall
- [ ] Refer for surgical ablation

26. If **all** PVs are reconnected, do you change your strategy?

- ◯ Yes
- ◯ No

27. If yes, what is your strategy ?

- ◯ Re-isolate the PV, then **STOP (no more options allowed)**
- ◯ Re-isolate the PV plus non-PV trigger ablation
- ◯ Re-isolate the PV plus substrate mapping and individualized ablation
- ◯ Re-isolate the PV plus GP ablation
- ◯ Re-isolate the PV plus ostial potential ablation
- ◯ Re-isolate the PV plus a more antral ablation
- ◯ Re-Isolate the PV and ablate empirical targets (see below)
- ◯ Post ablation Re-establish AAD

28. Empirical ablation targets beyond PVI, select all that apply:

- ◯ CTI
- ◯ PWI
- ◯ Roofline
- ◯ SVC
- ◯ LAA
- ◯ CS
- ◯ Septum
- ◯ Ligament / vein of Marshall
- ◯ Refer for surgical ablation

SECTION 5: Specific ablation strategies in Redo procedures with NO PV

reconnection

29. All PVs are isolated, what is your strategy?

- [ ] **STOP (no more options allowed)**
- [ ] Non-PV trigger ablation
- [ ] substrate mapping and individualized ablation
- [ ] GP ablation
- [ ] ostial potential ablation
- [ ] a more antral ablation
- [ ] Re-Isolate the PVs and ablate empirical targets (see below)
- [ ] Post ablation Re-establish AAD

30. Empirical ablation targets beyond PVI, select all that apply:

- [ ] I do not ablate empirical targets
- [ ] CTI
- [ ] PWI
- [ ] Roofline
- [ ] SVC
- [ ] LAA
- [ ] CS
- [ ] Septum
- [ ] Anterior line
- [ ] Ligament /vein of Marshall

EHRA European Heart Rhythm Association

Survey on strategies for repeat ablation for Atrial Fibrillation

Section 6: Failed rhythm control

31. What do you do in case of failed rhythm control with catheter ablation ± antiarrhythmic drugs?

- ( ) Transfer to a different Center
- ( ) Refer for surgical ablation
- ( ) Pharmacological rate control
- ( ) Pace and ablate strategy
- ( ) Other (please specify)

32. When do you consider rate control instead of rhythm control

- After 1 failed catheter ablation
- After 2 failed catheter ablations
- After 3 or more failed catheter ablations
- Never

33. Does your centre perform surgical ablation for AF?

- Yes
- No

34. When do you refer patients for surgical ablation?

- After 1 failed catheter ablation
- After 2 failed catheter ablations
- After 3 or more failed catheter ablations
- Never

35. If you have any comments or questions on the survey, please fill in the comment box and/or feel free to contact us at: sergioconti.md@gmail.com and dominik.linz@mumc.nl
